# Optimization of Coagulation-Flocculation Process in Efficient Arsenic Removal from Highly Contaminated Groundwater by Response Surface Methodology

**DOI:** 10.3390/molecules27227953

**Published:** 2022-11-17

**Authors:** Saba Amiri, Vahid Vatanpour, Tao He

**Affiliations:** 1 Department of Applied Chemistry, Faculty of Chemistry, Kharazmi University, Tehran 15719-14911, Iran; 2 National Research Center on Membrane Technologies, Istanbul Technical University, Maslak, 34469 Istanbul, Turkey; 3 Laboratory for Membrane Materials and Separation Technologies, Shanghai Advanced Research Institute, Chinese Academy of Sciences, Shanghai 201210, China

**Keywords:** arsenic removal, coagulation-flocculation, ferric chloride, Box-Ehnken design, groundwater treatment

## Abstract

Elevated arsenic (As) contamination in water, especially groundwater, has been recognized as a major problem of catastrophic proportions. This work explores As(V) removal via the coagulation-flocculation process by use of ferric chloride coagulant and polyacrylamide k16 co-coagulant as a first time. The effects of major operating variables such as coagulant dosing (50, 125 and 200 mg/L), co-coagulant dosing (5, 12.5 and 20 mg/L), pH (6, 7and 8), fast mixing time (1, 2 and 3 min), and fast mixing speed (110, 200 and 300 rpm) on As(V) removal efficiency were investigated by a Box-Behnken statistical experiment design (BBD) and response surface methodology (RSM). According to factors F values, coagulant dosing, rapid mixing speed, pH, and co-coagulant dosing showed the most effect on As(V) removal efficiency, and the rapid mixing time factor indicated the slightest effect. The proposed quadratic model was significant with a *p* value < 0.0001 and has satisfactorily described the experimental data with *R*^2^ and adjusted *R*^2^ values of 0.9855 and 0.9738, respectively. Predicted model optimal conditions with target of complete As(V) removal were coagulant dosing = 197.63 ppm, co-coagulant dosing = 19.55 ppm, pH = 7.37, fast mixing time = 1.43 min and fast mixing speed = 286.77 rpm. The treatment of Nazarabad well water sample with an initial As(V) concentration of 5 mg/L under the optimal conditions removed 100% As(V) with the volume of produced sludge of 10.7 mL/200 mL. Increasing coagulant dosing, co-coagulant dosing, fast mixing time and fast mixing speed operation parameters from low-level to high-level values indicated 78%, 20%, 10.52% and 9.47% increases in volume of the produced sludge, respectively. However, a reduction of 13.63% in volume of the produced sludge resulted via pH increases.

## 1. Introduction

Arsenic contamination in groundwater originating from anthropogenic phenomena or natural sources has been turned into a worldwide crisis [1]. Millions of people in different parts of the world are at risk of health problems through exposure to high levels of As via intake of As-rich groundwater [2]. Effects of long-term and excessive intake of As compounds result in serious arsenicosis, including skin lesions, neurological disorders, cardiovascular disease, and various types of cancers [3]. In addition, the risk of water source scarcity confirms the necessity of groundwater source maintenance in supplying safe drinking water with an increasing prospect. Therefore, groundwater treatment and mitigation of As pollution with the best available strategies have attracted many researchers’ special attention as the most challenging recent issues to work on [4,5,6,7]. To date, various available technologies include adsorption onto activated surfaces [8], ion-exchange [9], precipitation [10], coagulation-flocculation [11] and membrane separation [12]. All of the mentioned technologies are more efficient in As(V) removal from aqueous solutions [13]. Because As compounds mostly exist in oxyanions of trivalent arsenite (As(III)) or pentavalent arsenate (As(V)) species in natural groundwater, dispersion of its species is mainly dependent on pH conditions [14]. The dominant As(III) species are uncharged at the lower pH value of 9.2, while As(V) compounds possess a negative charge in natural waters within the pH range of 4–10 [15]. Therefore, As(V) species indicate a higher tendency to particle surface of many adsorbents and coagulants, which eventually favors adsorption and co-precipitation processes [16]. 

Among the existing As removal technologies, the coagulation-flocculation process is known as a prominent physico-chemical technique for As removal, which provides high removal efficiency even at high concentrations of As(V). This method indicates many advantages such as high stability, low cost, simple operation, and fast sedimentation [17]. It is reported that the coagulation-flocculation process, especially with ferric ion coagulant agents, is the best method for As(V) removal [18,19]. As(V) species possess a negative charge in natural waters above pH 2.2 and are electrostatically attracted to the positive charge on ferric hydroxide surfaces at ambient temperature, and fast sedimentation takes place via the Jar test [13].

The coagulation experiments were conducted through the Jar test, which simulates three steps including coagulation, flocculation, and settling [20,21]. The combination of a coagulant agent followed by a floc creation and consequently sedimentation of the produced sludge is a potential method applied for the efficient As removal from groundwater via the Jar test. Incorporation of the coagulant agents possessing a positive charge reduces the negative charge of the colloids, and accordingly, aggregation of these particles results in the formation of larger particles [22]. Soluble As is trapped by precipitating onto the growing flocs, and then, it is omitted from the aqueous environment with other precipitating species [23]. The existing forms of arsenic in water, initial As concentration, coagulants and the co-coagulant type and dosing, pH condition, time duration and speed of mixing during the flocculation step, and settling time were found as effective factors in As removal efficiency via coagulation-flocculation processes [23,24,25]. Commonly used ferric and aluminum-based coagulants such as ferric chloride or ferric sulfate and aluminum sulfate are usually applied in As removal through the coagulation-flocculation process due to cost-effectiveness and easy accessibility [23,26,27]. Performance of the Al salts coagulants such as aluminum chloride and two types of polyaluminum chloride was investigated in As(V) removal with an initial concentration of 280 ppb. All the three mentioned coagulants indicated a notable ability to decrease the As(V) concentration below the maximum contaminant level of 10 µg/L [28]. Better coagulating performance of the ferric salts compared with the aluminum salts has been confirmed due to their higher density of adsorption sites [29]. A higher rate of As(V) and Fe(III) precipitation with the formation of the insoluble compounds or surface complexation via adsorption of As(V) species on active sites of Fe hydroxide could intensify As(V) removal [30]. The removal efficiency of As(III) from real textile wastewater by the coagulation-flocculation process has been reported to be 81% under the optimum condition of 0.64 mg/L of FeCl_3_ coagulant and pH value of 8.1 with the flocculant volume of 2.6 mL/L [2]. In the more detailed study, As(V) removal efficiencies from arsenic-contaminated drinking water sources was studied with ferric chloride, ferric sulfate, and ferrous sulfate as the coagulant. The obtained results showed that higher As(V) removal efficiency was achieved with Fe(III) ions at the lower coagulant dose and pH value compared with Fe(II) ions [30]. As(V) removal could be improved by pH adjustment via regulating the distribution of coagulant species, and the obtained results showed that AlCl_3_ could benefit most from pH adjustments in terms of As(V) removal efficiency [28]. Based on paper reviews, an increasing trend in As(III) adsorption was reported with pH increasing while As(V) adsorption reduced with pH increasing [31]. The effective pH range for arsenic removal was recognized to be 5–7 and 5–8 for aluminum and ferric ions, respectively [32]. It was reported that As removal efficiency via FeCl_3_ was most probably affected by pH and Fe/As molar ratios. In the FeCl_3_ coagulation process, higher As(V) removal was obtained at a lower pH level compared with As(III) removal, and the opposite trend was concluded at a higher pH value. The crossover pH decreased gradually from 8.5 to 7.4 with an increasing As/Fe ratio from 0.12 to 0.50. Favored results of As(V) removal were obtained at low equilibrium concentration and pH value, and the opposite was obtained in As(III) removal [31]. Application of the organic polymers including natural and synthetic polyelectrolytes as the co-coagulant agent indicated favored results in contamination removal via the coagulation-flocculation process [33]. Higher removal of the toxic compounds and turbidity were obtained by the use of polyelectrolyte co-coagulants by improving the settling time step and reducing the sludge volumes. In addition to the mentioned advantages, it is reported that polyelectrolytes usage in the coagulation system may be effective at neutral pH, and metal coagulant dosage can be reduced without any reduction in removal efficiency [34]. The performance of the polyelectrolyte co-coagulant depends on its charge density and molecular weight [35]. Limited numbers of studies have been reported to investigate the effects of cationic [36,37,38,39], anionic [40], and nonionic [11] polyelectrolytes as co-coagulants in arsenic removal by the coagulation-flocculation process. In the flocculation step, the polyelectrolyte polymers resulted in the bridge formation between the larger mass particles and agglomerates, and trapping the As species in formed clumps accelerate the As precipitation [22]. Considering the effects of fast mixing time and fast mixing speed parameters along with co-coagulant combination in improving As removal efficiency are necessary to take into account [16]. As(V) removal through the coagulation-flocculation process has been reported to be more efficient than As(III) under similar experiment conditions [39,40]. To improve the As removal efficiency, oxidation pre-treatment was conducted to convert As(III) to As(V) prior to As removal by the coagulation-flocculation method [41,42,43,44]. The present work aims to study the removal efficiency of As(V) from arsenic-polluted groundwater wells coming from Nazarabad in Iran through the coagulation-flocculation process with ferric chloride coagulant. The Nazarabad well as a source of highly arsenic-polluted groundwater with an initial As(V) concentration of 0.5 mg/L was chosen to investigate the efficiency of the proposed approach for removal of arsenic (V) even at high concentration. As a novelty of the study, polyacrylamide K16 cationic polyelectrolyte was explored as a novel co-coagulant with ferric chloride coagulant in the removal of high concentrations of As(V) for the first time. The BBD design with the RSM method was used to optimize the important operating parameters of the coagulation-flocculation process including coagulant dosing, co-coagulant dosing, pH, fast mixing time and fast mixing speed to obtain the maximum As(V) removal efficiency. The regression quadratic model was developed to explain the relationship between the independent factors and the As(V) removal response. Furthermore, variation in produced sludge volumes as a determining characteristic inefficiency of the coagulation-flocculation method was studied related to each of the coagulant dosing, co-coagulant dosing, pH, fast mixing time, and fast mixing speed factors. The findings provide new insight into the availability of BBD in As(V) removal by the coagulation-flocculation process and better knowledge of the effective parameters to achieve complete As(V) removal efficiency from the well water sample.

## 2. Results and Discussion

### 2.1. Model Fitting and Statistical Analysis

Response surface methodology was applied to model the experimental data of the As(V) removal process obtained from the five-factor Box-Behnken design, including coagulant dosing, co-coagulant dosing, pH, fast mixing time, and fast mixing speed. For model development and regression analysis, linear, quadratic, and cubic models were studied and correlated with the experimental data. Adequacy of each model in the prediction of As(V) removal was investigated through model statistics. Model summary statistics study for the response variables investigated is shown in Table S1. As it is obvious, the linear model with the predicted *R*^2^ (Pre- *R*^2^) and adjusted *R*^2^ (Adj *R*^2^) values of 0.8052 and 0.8309, respectively, seems to be inadequate for the experimental data. The quadratic model was proposed for better analysis of As(V) removal efficiency, as its better fitting of the experimental data with low standard deviations, the highest correlation coefficients, Adj *R*^2^, Pre *R*^2^ values, and the lowest *p* values without aliasing occurred in the cubic model due to insufficient points to estimate the model coefficients. Therefore, the quadratic model was chosen for further analysis [45].

### 2.2. Analysis of Variance

Table 1 presents the results of the analysis of variance (ANOVA) as well as the quadratic models’ statistics. The ANOVA data indicated that this regression model was highly significant (*p* value < 0.0001) with an F value of 84.71. There is only a 0.01% chance that an F value this large could occur due to the noise. Additionally, the “Lack of fit F value” of 1.36 implies that the “Lack of fit” is not significant relative to pure errors. There is a 39.39% chance that the “Lack of fit F value” this large could occur due to noise. According to the F values reported in the ANOVA table, among the studied factors, the coagulant dosing factor (A) with the highest F value is the most effective factor in the As(V) removal process. Then, rapid mixing speed, pH, and co-coagulant dosing factors are effective factors in arsenic (V) removal, and the factor of rapid mixing time with the lowest F value indicated a slight effect on the response.

The coefficient of determination *R*^2^ with a high value of 0.9855 showed a good agreement between the model prediction and the experimental results. The Adj *R*^2^ parameter permitting for the degrees of freedom associated with the sums of the squares is also taken into account in the lack-of-fit test. The Adj *R*^2^ parameter as an indicator of the measured variation about the mean indicated the approximate value of *R*^2^ at 0.9738. The Pre-*R*^2^ of 0.9476 is in reasonable agreement with the Adj *R*^2^ of 0.9738, i.e., the difference is less than 0.2. In the present study, adequate precision of 37.7945 indicates that the model is acceptable for navigation of the design space [46]. The final regression model in terms of the actual values for the quadratic type can be expressed using Equation (1).

Y = −701.43 + 0.74X_1_ + 0.41X_2_ + 192.64X_3_ + 18.14X_4_ + 0.21X_5_ + 0.002X_1_X_2_ + 0.006X_1_X_3_ − 0.01X_1_X_4_ + 0.00003X_1_X_5_ + 0.03X_2_X_3_ − 0.20X_2_X_4_ − 1.64E − 18X_2_X_5_ − 0.25X_3_X_4_ − 0.02X_3_X_5_ + 3.12E − 17X_1_X_5_ − 0.001X_1_^2^ − 13.72X_3_^2^ − 3.22X_4_^2^ − 0.00006X_5_^2^ (1)

**Figure 1:**
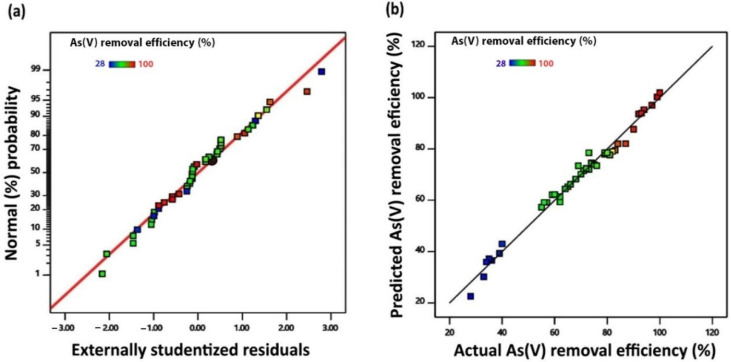
(**a**) Normal plot of residuals. (**b**) Predicted versus actual plot of As(V) removal efficiency.

The model predictability indicating adequate approximation of the real system is confirmed via diagnostic plots including the normal plot of residuals and the plot of predicted value versus actual value. The normal probability plot of the studentized residuals in the As(V) removal process as presented in Figure 1a follows a normal distribution without any obvious pattern, and some scattered data points can generally be expected. Moreover, the predicted values are in good agreement with the experimental values, which indicates that the proposed model describes the studied system well, as shown in Figure 1b. The obtained results of the analysis are in agreement with the given *R*^2^ value and confirmed that the quadratic model can estimate the experimental data points with high accuracy.

### 2.3. Three-Dimensional Surface and Two-Dimensional Contour Plots of As(V) Removal

**Figure 2:**
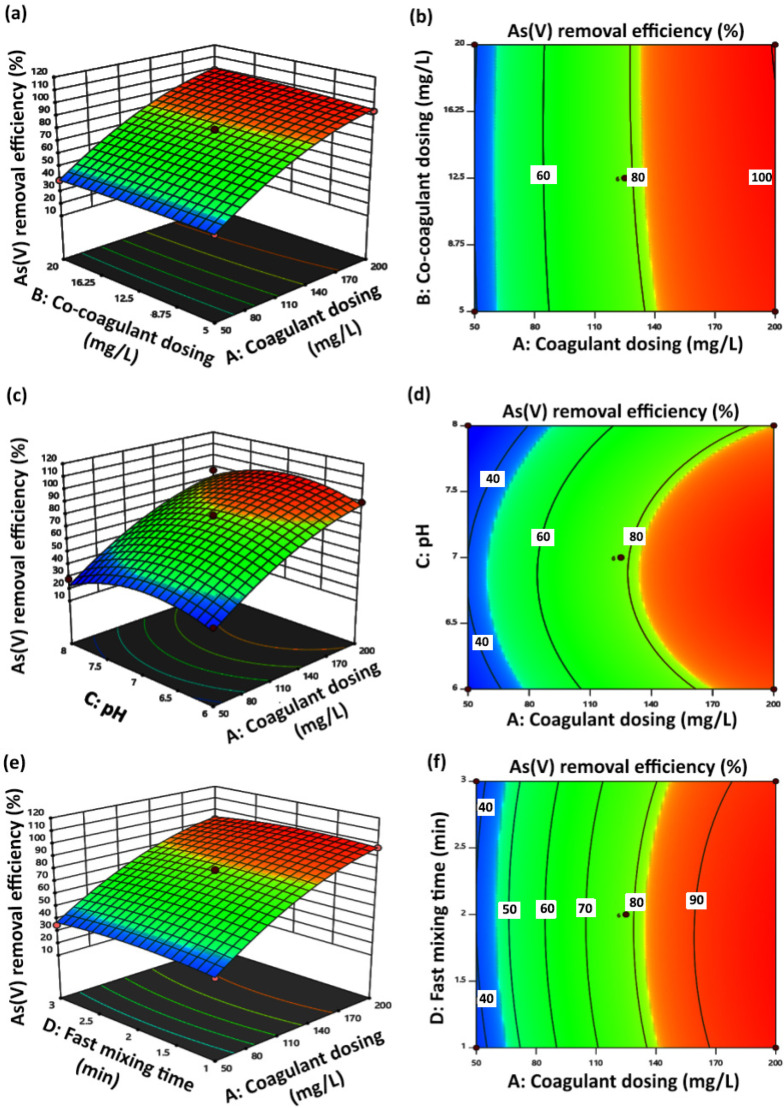

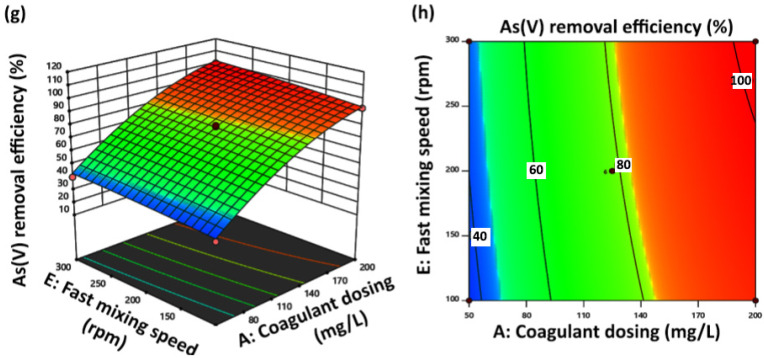
Three-dimensional surface and two-dimensional contour plots of As(V) removal. (**a**,**b**) show the effect of coagulant dosing (mg/L) and co-coagulant dosing (mg/L); (**c**,**d**) show the effect of coagulant dosing (mg/L) and pH; (**e**,**f**) show the effect of coagulant dosing (mg/L) and fast mixing time (min); (**g**,**h**) show the effect of coagulant dosing (mg/L) and fast mixing speed (rpm).

A three-dimensional (3D) response surface plot displays the function of two factors on the response, while all the other factors remain unchanged at zero-level values. Thus, the main effects, as well as the interaction effects of the two factors, can be investigated. In addition to 3D plots, contour maps can indicate the influence of the design parameters on the response. Therefore, the influence of the experimental variables on the As(V) removal efficiency was studied by the generation of 3D response surface plots and contour maps based on the model equation. Figure 2a,b displays the 3D response surface plot and contour map of coagulant dosing (A) and co-coagulant dosing (B) factors at zero-level values of pH (C), fast mixing time (D), and fast mixing speed (E) factors. Coagulant dosing factor (A) with the highest F value is the most effective factor in the As(V) removal process. As(V) removal efficiency increased with the increase in coagulant dosing (A) from 50 up to 200 ppm. On the other hand, As(V) removal efficiency was increased by increasing co-coagulant dosing (B) from 5 up to 12.5 ppm and remained unchanged up to 20 ppm of the co-coagulant dosing (B). By considering coagulant dosing (A) and co-coagulant dosing (B) interaction factors, complete As(V) removal occurred at 200 and 12.5 ppm concentrations of ferric chloride coagulant dosing and polyacrylamide k16 co-coagulant dosing, respectively, and zero-level values of the other mentioned factors. Three-dimensional response surface and two-dimensional contour plot displaying the relationship between coagulant dosing (A) and pH (C) at the center level of co-coagulant dosing (B), fast mixing time (D), and fast mixing speed (E) factors are presented in Figure 2c,d, respectively. pH interaction effect investigation at the range of 6–8 indicated that efficiency of As(V) removal increased with the increase in pH value from 6 up to 7, and maximum As(V) removal efficiency was obtained at the pH value of 7. Reduction in As(V) removal efficiency with further increments in pH value up to 8 was related to competition of the present species to be adsorbed by positively charged centers. As(V) species become more negatively charged with increasing pH, and on the other hand, the surface of precipitated Fe(III) particles decreases with increasing pH value. Thus, the lowest percentage of As(V) removal was reported due to the reduction of removal ability of ferric chloride coagulant at pH = 8 [43]. The highest percentage of As(V) removal was obtained at the coagulant dosing of 200 ppm and pH = 7, as can be seen from Figure 2c.

As it was mentioned in the previous part, according to ANOVA data reports, the fast mixing time (D) factor with the lowest *p* value was introduced as the least effective independent factor in experiments designed for the coagulation-flocculation process to remove As(V). Three-dimensional and contour plots of coagulant dosing (A) and fast mixing time (D) interaction factors at center level values of the other factors are displayed in Figure 2e,f, respectively. Changes in the fast mixing time factor (D) from 1 to 3 min did not indicate a significant effect on As(V) removal efficiency. At 2 min of fast mixing time (D) and 200 mg/L of coagulant dosing (A), complete As(V) removal efficiency was reported. The percentage of As(V) removal increased with increasing the fast mixing speed from 100 to 300 rpm along with increasing coagulant dosing at the center levels of the other effective factors, as is concluded from the 3D response surface and contour plots displayed in Figure 2e,f, respectively. Increasing the fast mixing speed of the As(V) removal process through coagulation-flocculation provides more collisions between the present As(V) species in the solution and the ferric chloride coagulant and k16 cationic co-coagulant. It was estimated that at the maximum fast mixing speed value of 300 rpm, the formed macroflates are broken down to form smaller-dimension flocs, which provide more surface area to trapped As(V) species through the generation of cluster flocs. Therefore, As(V) removal efficiency improvement at the high level of fast mixing speed factor (E) can be justified by increasing the collision of the particles, which increases the adsorption level of As(V) species [47].

### 2.4. Optimal Coagulation-Flocculation Condition in As(V) Removal 

**Figure 3:**
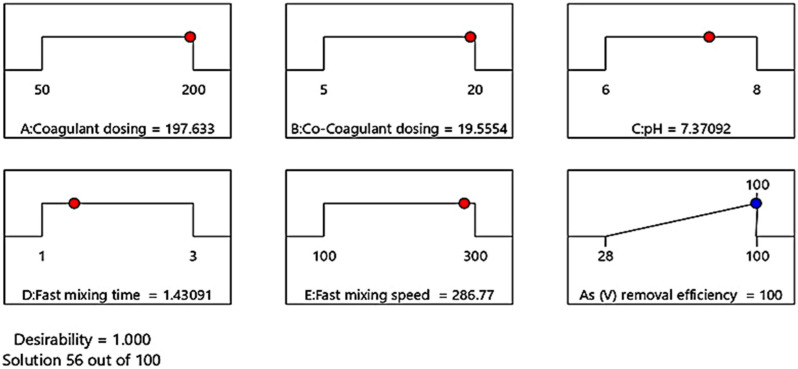
The optimal condition of As(V) removal process with the coagulation-flocculation method.

The optimization of the coagulation-flocculation process via BBD includes studying the response of the statistically designed combinations, evaluating the coefficients by fitting them in a mathematical model that best fits the experimental conditions, predicting the response of the fitted model, and checking the adequacy of the model [48]. To determine the optimal conditions of the coagulation-flocculation process for complete removal of As(V) with the initial concentration of 10 ppm, the effective parameters were optimized using DX11 Design-Expert software. For this purpose, the values of the effective factors were kept in the levels range, and the target of 100% As(V) removal efficiency was set. The software suggested several solutions under the different conditions of effective factors resulting in complete As(V) removal efficiency. Selected optimal conditions for complete As(V) removal with desirability of 1 is shown in Figure 3. Under these conditions, in the presence of ferric chloride coagulant and k16 co-coagulant with concentrations of 197.63 and 19.55 ppm, respectively, at pH = 7.37, which is similar to the pH value of the studied well water sample and fast mixing speed of 286.77 rpm, complete As(V) removal with the initial concentration of 10 ppm occurred within 1.43 min. 

To confirm the model prediction, the As(V) removal process was repeated under the optimal conditions with the Nazarabad well water sample containing the specific As(V) concentration of 5 mg/L with three repetitions. Under the suggested optimal conditions of the model, the experimental result of the complete (100%) removal of As(V) from the real well water sample was obtained, which confirms high accuracy of the proposed model based on the response surface method for predicting the optimal conditions of As(V) removal. 

### 2.5. Volume of the Produced Sludge Analysis

**Figure 4:**
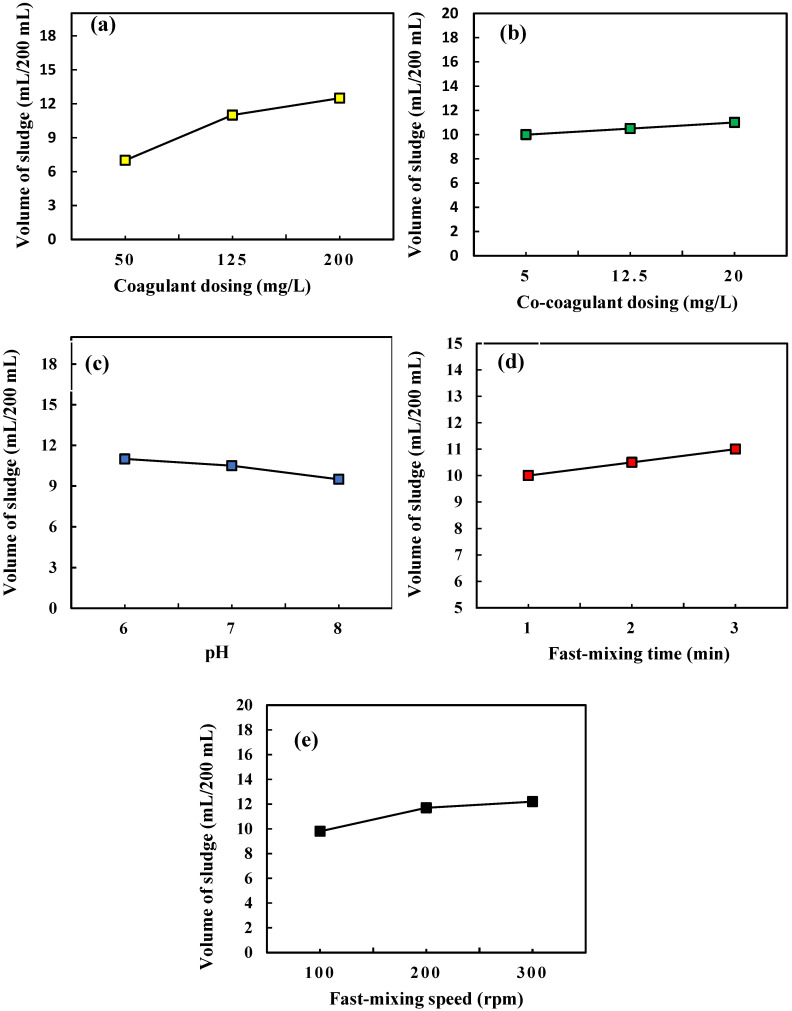
The variations of the produced sludge volume in coagulation-flocculation of As(V) removal related to (**a**) coagulant dosing, (**b**) co-coagulant dosing, (**c**) pH, (**d**) fast mixing time, and (**e**) fast mixing speed parameters.

The management of the produced sludge volume in the coagulation-flocculation process as a major portion of the wastewater treatment cost is of high importance [49]. The amount of the produced sludge is an important index to evaluate the coagulation-flocculation efficiency [47,50]. The variations in the sludge volume related to coagulant dosing, co-coagulant dosing, and pH, fast mixing time, and fast mixing speed parameters are displayed in Figure 4a–e, respectively. In each diagram, the variation trend of the sludge is plotted in the center values of the other variables. As can be seen in Figure 4a,b, the volume of the produced sludge has increased with increasing coagulant dosing and co-coagulation dosing. In the As(V) removal process of 200 mL, aqueous solution with an As(V) concentration of 10 mg/L, by the use of 50, 125, and 200 mg/L of ferric chloride coagulant, reported volumes of the produced sludge at 7, 11 and 12.5 mL, respectively. The volume of the produced sludge varied from 10 to 12 mL with increasing co-coagulant dosing from 5 up to 20 mg/L. Investigation of the solution pH effect on the volume of the produced sludge confirmed a 13.63% reduction in the volume of the produced sludge by increasing the solution pH from 6 up to 8, as presented in Figure 4c. Reduction in sludge volume by increasing the solution pH can be attributed to the increase in the negative charge of the present As(V) species in the solution and the decrease in the level of the precipitated Fe(III) particles, resulting in a reduction of the produced sludge volume [51]. Increasing the fast-mixing time and fast mixing speed variables resulted in increases in the volume of the produced sludge as shown in Figure 4d,e, respectively. Increasing the fast-mixing time from 1 to 3 min provided more opportunity for the formation of colloidal particles and clots and indicated a 10.52% growth in the volume of produced sludge. The possibility of collisions between the As(V) species and Fe(III) ions was increased with increasing the fast mixing speed of the coagulation-flocculation process, which resulted in a higher rate of As(V) trapping due to improving the bridging property of the coagulated particles [52,53]. In the coagulation-flocculation process of As(V) removal from the Nazarabad well water sample, complete As(V) removal efficiency occurred with the production of 10.7 mL sludge under the investigated optimum conditions. These results illustrate the advantages of the usage of ferric chloride coagulant and polyacrylamide k16 co-coagulant and optimization of the coagulation-flocculation effective factors, which resulted in As(V) removal efficiency improvement as well as a reduction in the amount of the produced sludge.

### 2.6. Comparison with Literature Data

Table 2 presents a comparison of our work with the other studies related to As(V) removal through the coagulation-flocculation method. As can be seen from Table 2, compared with the other studies, our work provides complete As(V) removal efficiency at a high concentration of 5 mg/L with lower consumptions of 197.3 and 19.5 mg/L for FeCl_3_ and polyacrylamide k16 as the coagulant and co-coagulant, respectively. Comparison of our work results with the reported literature data by considering the dosages and concentrations of the used coagulant and co-coagulant agents confirms that although the consumption of the coagulant and co-coagulant dosages was higher, the initial concentration of As(V) in our study was hundreds of times higher than the other works, and we obtained a removal efficiency of 100% for a high concentration of As(V).

## 3. Experimental Section

### 3.1. Materials and Apparatus

Ferric chloride hexahydrate powder (FeCl_3_·6H_2_O), sodium arsenate (Na_3_ASO_4_), sodium hydroxide (NaOH), hydrochloric acid (HCl), potassium iodide (KI), ammonium molybdate ((NH_4_)_6_MO_7_O_24_·4H_2_O), and ethyl violet (C_31_H_42_N_3_Cl) were purchased from Sigma-Aldrich, USA. Polyacrylamide k16 cationic–polyelectrolyte (Molecular weight: 6 × 10^6^, Ionization degree: 32%, Mesh size: 20–80, UL viscosity: 6.4) was provided from XiaChem (China). Characterization of the arsenic polluted groundwater well used in the study coming from Nazarabad in Iran is presented in Table 3.

All As(V) aqueous solutions were prepared using distilled water, and UV–vis spectrophotometer (Perkin Elmer, Lambda 850, Rocklin, CA, USA) was used to determine their concentrations. The coagulation-flocculation experiments were carried out in a multiple stirrer jar test apparatus (Pars Teb Novin, international. co, Tehran, Iran). A pH/redox/temperature meter (Sana, SL 901, Tehran, Iran) was applied to adjust the pH of the prepared solution.

### 3.2. As(V) Removal Procedure

Jar test analysis as a batch pilot system followed by sedimentation according to the ASTM2035 standard method was conducted in As(V) removal [56]. In doing so, a stock solution of Na_3_AsO_4_ salt at As(V) concentration of 1 g/L was prepared using distilled water, and then, each jar was filled with 200 mL As(V) solution with the concentration of 10 mg/L prepared with diluting the stock solution. After adjusting the solution pH at the range of 6–8 by the addition of HCl or NaOH, distinct values of ferric chloride at three concentrations of 50, 100, and 200 mg Fe/L were added. After the addition of polyacrylamide K16 co-coagulant at dosages of 5, 10, and 20 mg/L, the samples were mixed rapidly at rates of 100, 200, and 300 rpm for 1, 2, and 3 min, based on designed conditions. After the fast mixing step, the mixing speed was reduced, and the solutions were subjected to slow mixing at 50 rpm for 20 min. After this period, the stirrer was stopped, and the flock was allowed to settle for 30 min [2]. Finally, water sampling was conducted from 2 cm below the supernatants to determine the As(V) concentration by the spectrophotometric method by measuring the absorbance of the resulted blue complex of molybdoarsenate and ethyl violet at 612 nm [46]. The percentage of As(V) removal efficiency was calculated as follows [57]:

(2) $$R(\%)=\frac{C_{0}-C}{C_{0}}\times 100$$

where *C*_0_ and *C* are the initial and final concentration values of As(V), respectively.

To enhance the work precision, all the experiments were conducted at room temperature (25 ± 2 °C) in triplicate under identical conditions, and the average results of three measurements were reported. To evaluate the effect of each of the coagulation-flocculation variables on produced sludge volume, the volume of the produced sludge in each experiment was measured after 30 min of settling.

### 3.3. Experimental Design and Data Analysis

The BBD as one of the most common designs of the principal response surface methodology provides useful data for optimization of different chemical and physical processes with reasoning design and excellent outcomes [48].

The three-level-five-variable BBD was employed to study the effect of the major operating factors on the As(V) removal efficiency and to determine the combination of the effective factors resulting in complete As(V) removal efficiency. For As(V) removal with the coagulation-flocculation process, according to the principle of BBD, three-level-five important operation variables of the process including coagulant dosing (X_1_), co-coagulant dosing (X_2_), pH (X_3_), fast mixing time (X_4_), and fast mixing speed (X_5_) were considered as the independent variables in 3 levels of −1, 0, and +1 [58]. The minimum and maximum ranges of the five factors in the terms of uncoded and coded symbols in three levels are illustrated in Table 4.

The Box-Behnken design matrix of the As(V) removal experiment with 5 mentioned factors by use of Design-Expert software version 11.1.0.1 (StatEase, Inc. Minneapolis, MN, USA) is shown in Table S2. All 46 designed trials were performed in triplicate, and the averages of As(V) removal efficiency (Y) were taken as the response. 

Based on the obtained results, the response function that correlates the relationship between independent variables and As(V) removal efficiency response is expressed using polynomial regression equations as follows:

(3) $$Y=\beta_{0}+\sum_{i=1}^{k}\beta_{i}X_{i}+\sum_{i=1}^{k}\beta_{\mathrm{ii}}X_{i}^{2}+\sum_{i=1}^{k-1}\sum_{j=i+1}^{k}\beta_{\mathrm{ij}}X_{i}X_{j}+\varepsilon$$

where Y is the predicted response; k is the number of the independent factors; X_i_, and X_j_ are the independent factors as coded values, which influence the predicted response Y; *β*_0_ is the constant coefficient; *β*_i_, *β*_ij_, and *β*_ii_ are the coefficients of linear, interaction, and quadratic term, respectively, and *ε* represents the residual error. 

## 4. Conclusions

A three-level-five-variable BBD was studied for As(V) removal from groundwater via the coagulation-flocculation process by use of ferric chloride coagulant and polyacrylamide k16 co-coagulant. Coagulant dosing (50, 100 and 200 mg/L), co-coagulant dosing (5, 10, and 20 mg/L), pH (6, 7 and 8), fast mixing time (1, 2, and 3 min), and fast mixing speed (100, 200 and 300 rpm) were considered as major operating variables on the As(V) removal efficiency. The proposed quadratic model was significant with a *p* value < 0.0001 and has satisfactorily described the experimental data with *R*^2^ and adjusted *R*^2^ values of 0.9855 and 0.9738, respectively. The obtained optimal conditions with the target of 100% As(V) removal efficiency were coagulant dosing of 197.63 ppm, co-coagulant dosing of 19.55 ppm, pH value of 7.37, and fast mixing time and fast mixing speed of 1.43 min, and 286.77 rpm, respectively. Increasing coagulant dosing, co-coagulant dosing, fast mixing time and fast mixing speed operation parameters from low-level to high-level values indicated 78%, 20%, 10.52% and 9.47% increases in volume of the produced sludge, respectively. However, a reduction of 13.63% in volume of the produced sludge resulted via pH increasing. The treatment of the Nazarabad well water sample with an initial As(V) concentration of 5 mg/L under the proposed model optimal conditions could remove 100% As(V) with a volume of produced sludge of 10.7 mL. This work may contribute to a better understanding and prediction of the application of ferric chloride coagulant and polyacrylamide k16 cationic–polyelectrolyte co-coagulant in As(V) removal from aqueous environments, to reduce potential risks to humans.

## Figures and Tables

**Table 1:** Analysis of variance of response surface quadratic model for As(V) removal via the coagulation-flocculation method.

Source	Sum of Squares	df	Mean Square	F Value	*p* Value	
Model	16,377.99	20	818.90	84.71	<0.0001	significant
A: Coagulant dosing	13,689.00	1	13689.00	1416.10	<0.0001	
B: Co-Coagulant dosing	25.00	1	25.00	2.59	0.1204	
C: pH	175.56	1	175.56	18.16	0.0003	
D: Fast mixing time	7.56	1	7.56	0.7823	0.3849	
E: Fast mixing speed	225.00	1	225.00	23.28	<0.0001	
AB	6.25	1	6.25	0.6466	0.4289	
AC	1.0000	1	1.00	0.1034	0.7504	
AD	4.00	1	4.00	0.4138	0.5259	
AE	0.2500	1	0.2500	0.0259	0.8735	
BC	0.2500	1	0.2500	0.0259	0.8735	
BD	9.00	1	9.00	0.9310	0.3438	
BE	0.0000	1	0.0000	0.0000	1.0000	
CD	0.2500	1	0.2500	0.0259	0.8735	
CE	20.25	1	20.25	2.09	0.1602	
DE	0.0000	1	0.0000	0.0000	1.0000	
A^2^	730.00	1	730.00	75.52	<0.0001	
B^2^	5.76	1	5.76	0.5960	0.4473	
C^2^	1645.00	1	1645.00	170.17	<0.0001	
D^2^	91.00	1	91.00	9.41	0.0051	
E^2^	3.64	1	3.64	0.3766	0.5450	
Residual	241.67	25	9.67			
Lack of Fit	204.17	20	10.21	1.36	0.3939	not significant
Pure Error	37.50	5	7.50			
Cor Total	16,619.65	45				

*R*² = 0.9855, Adj *R*² = 0.9738, Pre-*R*^2^ = 0.9476, Adeq precision = 37.7945.

**Table 2:** A comparison of As(V) removal efficiency through coagulation-flocculation methods with different coagulants and co-coagulants.

Coagulant		Co-Coagulant		Initial [As(V)]	As(V) Removal Efficiency (%)	Ref.
Type	Dosage	Type	Dosage			
Al_2_(SO_4_)_3_	40 mg L^−1^	Magnafloc LT22	2 mg L^−1^	50 μg L^−1^	81	[11]
		Magnafloc LT27	2 mg L^−1^		80	
		Magnafloc LT20	2 mg L^−1^		76	
FeCl_3_	30 mg L^−1^	-	-	50 μg L^−1^	80	[33]
Fe_2_(SO_4_)_3_	40 mg L^−1^	-	-		72	
FeSO_4_	60 mg L^−1^	-	-		72	
Fe_2_(SO_4_)_3_	500 mg L^−1^	Activated silica Activated carbon Cationic polyacrylamide Polyvinyl alcohol Polyacrylic acid Anionic polyacrylamide	1.9 mg L^−1^ 1.6 g L^−1^ 50 mg L^−1^ 20 mg L^−1^ 5 mg L^−1^ 25 mg L^−1^	50 μg L^−1^	87 77 95 96 93 95 99	[54]
FeCl_3_	0.64 g L^−1^	Himoloc DR3000	2.6 g L^−1^	20 μg L^−1^	81.76	[2]
Hydrated lime/FeCl_3_ Hydrated lime/Al_2_(SO_4_)_3_	1000 mg L^−1^ FeCl_3_ 1000 mg L^−1^ Al_2_(SO_4_)_3_	- -	- -	9.8 mg L^−1^	98.9 81.6	[55]
FeCl_3_	197.3 mg L^−1^	Polyacrylamide k16	19.5 mg L^−1^	5 mg L^−1^	100%	This work

**Table 3:** Characterization of arsenic polluted groundwater well used for the experiment.

Components	Value/Concentration
pH	7.7
Conductivity (µS/cm)	1983
Alkalinity (mg/L as HCO_3_^−^)	277.6
TDS (mg/L)	1338
As(V) (mg/L)	0.5
Cl^−^ (mg/L)	492
NO_3_^−^ (mg/L)	8.3
SO_4_^2−^ (mg/L)	158
Na (mg/L)	2.39
Ca (mg/L)	84.52
Mg (mg/L)	185
Al (mg/L)	0.12
Fe (mg/L)	0.09
Mn (mg/L)	<0.01
Pb (µg/L)	30.7
Ni (µg/L)	22.1
Cr (µg/L)	4.5

**Table 4:** Levels of effective variables in Box-Behnken design of As(V) removal via coagulation-flocculation.

Variables	Symbols		Level −1	Level 0	Level +1
	Uncoded	Coded			
A: Coagulant dosing (mg/L)	X_1_	A	50	125	200
B: Co-Coagulant dosing (mg/L)	X_2_	B	5	12.5	20
C: pH	X_3_	C	6	7	8
D: Fast mixing time (min)	X_4_	D	1	2	3
E: Fast mixing speed (rpm)	X_5_	E	100	200	300

## Data Availability

All data are provided in the manuscript.

## Supplementary Materials

The following supporting information can be downloaded at https://www.mdpi.com/article/10.3390/molecules27227953/s1, Table S1: Model summary statistics for response variables investigated, Table S2: Experimental design and results of As(V) removal.

## Sample Availability

Samples of the compounds are not available from the authors.
