# Supplementary material for: Optimization of Coagulation-Flocculation Process in Efficient Arsenic Removal from Highly Contaminated Groundwater by Response Surface Methodology
Source: Molecules. 2022 Nov 17;27(22):7953. doi: 10.3390/molecules27227953 (PMC9693969; doi:10.3390/molecules27227953)
Supplement: Supplementary file 1 [file molecules-27-07953-s001.zip › molecules-1998700-supplementary.pdf]

Optimization of coagulation-flocculation process in efficient arsenic removal from highly contaminated groundwater by response surface methodology

Saba Amiri [a], Vahid Vatanpour [a,b,*], Tao He [c]

[a] Department of Applied Chemistry, Faculty of Chemistry, Kharazmi University, 15719-14911 Tehran, Iran

[b] National Research Center on Membrane Technologies, Istanbul Technical University, Maslak, 34469 Istanbul, Turkey

[c] Laboratory for Membrane Materials and Separation Technologies, Shanghai Advanced Research Institute, Chinese Academy of Sciences, Shanghai 201210, China

*** Corresponding author:** vahidvatanpour@khu.ac.ir

Table S1. Experimental design and results of As(V) removal

Run	A	B	C	D	E	Y: As(V) removal efficiency (%)
1	0.000	0.000	-1.000	-1.000	0.000	65
2	-1.000	0.000	0.000	0.000	1.000	40
3	0.000	1.000	-1.000	0.000	0.000	68
4	0.000	0.000	0.000	1.000	1.000	81
5	1.000	0.000	-1.000	0.000	0.000	90
6	-1.000	0.000	0.000	-1.000	0.000	36
7	0.000	0.000	0.000	0.000	0.000	80
8	0.000	-1.000	-1.000	0.000	0.000	66
9	0.000	0.000	1.000	0.000	-1.000	62
10	0.000	-1.000	0.000	1.000	0.000	75
11	-1.000	-1.000	0.000	0.000	0.000	39
12	0.000	0.000	0.000	-1.000	-1.000	71
13	0.000	-1.000	1.000	0.000	0.000	56
14	-1.000	0.000	0.000	1.000	0.000	35
15	0.000	0.000	-1.000	0.000	-1.000	62
16	0.000	0.000	0.000	0.000	0.000	79
17	1.000	0.000	0.000	0.000	1.000	100
18	0.000	0.000	-1.000	0.000	1.000	69
19	0.000	1.000	0.000	0.000	1.000	84
20	1.000	0.000	0.000	1.000	0.000	92
21	0.000	1.000	1.000	0.000	0.000	59
22	1.000	0.000	0.000	-1.000	0.000	97
23	0.000	0.000	0.000	0.000	0.000	73
24	1.000	0.000	0.000	0.000	-1.000	93
25	0.000	0.000	0.000	0.000	0.000	79
26	0.000	-1.000	0.000	-1.000	0.000	72
27	1.000	0.000	1.000	0.000	0.000	87
28	0.000	0.000	-1.000	1.000	0.000	64
29	0.000	0.000	0.000	0.000	0.000	80
30	0.000	0.000	1.000	-1.000	0.000	57
31	1.000	-1.000	0.000	0.000	0.000	94
32	-1.000	1.000	0.000	0.000	0.000	39
33	0.000	0.000	0.000	0.000	0.000	80
34	1.000	1.000	0.000	0.000	0.000	99
35	0.000	0.000	1.000	1.000	0.000	55
36	-1.000	0.000	1.000	0.000	0.000	28
37	0.000	-1.000	0.000	0.000	1.000	83
38	0.000	0.000	1.000	0.000	1.000	60
39	-1.000	0.000	0.000	0.000	-1.000	34
40	0.000	1.000	0.000	-1.000	0.000	79
41	0.000	0.000	0.000	1.000	-1.000	70
42	0.000	-1.000	0.000	0.000	-1.000	73
43	0.000	1.000	0.000	0.000	-1.000	74
44	-1.000	0.000	-1.000	0.000	0.000	33
45	0.000	1.000	0.000	1.000	0.000	76
46	0.000	0.000	0.000	-1.000	1.000	82

Table S2. Model summary statistics for response variables investigated.

Source	Std. Dev.	Sequential p-value	Lack of Fit p-value	R^2	Adjusted R^2	Predicted R^2	Remarks
Linear	7.90	< 0.0001	0.0099	0.8497	0.8309	0.8052	
2FI	9.05	1.000	0.0049	0.8522	0.7783	0.6542	
Quadratic	3.11	< 0.0001	0.3939	0.9855	0.9738	0.9476	Suggested
Cubic	4.03	0.9752	0.1065	0.9902	0.9561	0.5162	Aliased
